# Positive Influence of Being Overweight/Obese on Long Term Survival in Patients Hospitalised Due to Acute Heart Failure

**DOI:** 10.1371/journal.pone.0117142

**Published:** 2015-02-24

**Authors:** Simona Littnerova, Jiri Parenica, Jindrich Spinar, Jirí Vitovec, Ales Linhart, Petr Widimsky, Jiri Jarkovsky, Roman Miklik, Lenka Spinarova, Kamil Zeman, Jan Belohlavek, Filip Malek, Marian Felsoci, Jiri Kettner, Petr Ostadal, Cestmir Cihalik, Jiri Spac, Hikmet Al-Hiti, Marian Fedorco, Richard Fojt, Andreas Kruger, Josef Malek, Tereza Mikusová, Zdenek Monhart, Stanislava Bohacova, Lidka Pohludkova, Filip Rohac, Jan Vaclavik, Dagmar Vondrakova, Klaudia Vyskocilova, Miroslav Bambuch, Ladislav Dusek

**Affiliations:** 1 Institute of Biostatistics and Analysis, Faculty of Medicine, Masaryk University, Brno, Czech Republic; 2 Department of Cardiology, University Hospital Brno, Brno, Czech Republic; 3 Medical Faculty, Masaryk University, Brno, Czech Republic; 4 Department of Cardiovascular Disease, International Clinical Research Center, University Hospital St Anne’s, Brno, Czech Republic; 5 First Department of Cardiovascular Internal Medicine, University Hospital St Anne’s, Brno, Czech Republic; 6 2nd Department of Cardiovascular Internal Medicine, First Medical Faculty, Charles University, Prague and General Teaching Hospital of Prague, Prague, Czech Republic; 7 Kralovske Vinohrady University Hospital and the 3rd Faculty of Medicine, Charles University, Prague, Czech Republic; 8 Department of Internal Medicine, Hospital Frydek-Mistek, Frydek-Mistek, Czech Republic; 9 Department of Cardiology, Na Homolce Hospital, Prague, Czech Republic; 10 Department of Cardiology, Institute of Clinical and Experimental Medicine, Prague, Czech Republic; 11 Department of Internal Medicine, University Hospital Olomouc, Olomouc, Czech Republic; 12 2nd Department of Internal Medicine, University Hospital St Anne’s, Brno, Czech Republic; 13 Department of Internal Medicine, Hospital Havlickuv Brod, Havlickuv Brod, Czech Republic; 14 Department of Internal Medicine, Hospital Znojmo, Znojmo, Czech Republic; 15 Department of Cardiology, T.Bata Hospital Zlin, Zlin, Czech Republic; I2MC INSERM UMR U1048, FRANCE

## Abstract

**Background:**

Obesity is clearly associated with increased morbidity and mortality rates. However, in patients with acute heart failure (AHF), an increased BMI could represent a protective marker. Studies evaluating the “obesity paradox” on a large cohort with long-term follow-up are lacking.

**Methods:**

Using the AHEAD database (a Czech multi-centre database of patients hospitalised due to AHF), 5057 patients were evaluated; patients with a BMI <18.5 kg/m^2^ were excluded. All-cause mortality was compared between groups with a BMI of 18.5–25 kg/m^2^ and with BMI >25 kg/m^2^. Data were adjusted by a propensity score for 11 parameters.

**Results:**

In the balanced groups, the difference in 30-day mortality was not significant. The long-term mortality of patients with normal weight was higher than for those who were overweight/obese (HR, 1.36; 95% CI, 1.26–1.48; p<0.001)). In the balanced dataset, the pattern was similar (1.22; 1.09–1.39; p<0.001). A similar result was found in the balanced dataset of a subgroup of patients with *de novo* AHF (1.30; 1.11–1.52; p = 0.001), but only a trend in a balanced dataset of patients with acute decompensated heart failure.

**Conclusion:**

These data suggest significantly lower long-term mortality in overweight/obese patients with AHF. The results suggest that at present there is no evidence for weight reduction in overweight/obese patients with heart failure, and emphasize the importance of prevention of cardiac cachexia.

## Introduction

The overall mortality based on data from 19 prospective studies for a non-heart failure population of 1.46 million Caucasians was lowest in the group with a body mass index (BMI; in kg/m^2^) of 20.0–24.9 [1]; for the Asian population (1.1 million persons recruited in 19 cohorts) the lowest risk of death was seen for a BMI range of 22.6–27.5 [2]. Recent analyses in 57 prospective studies (89,4576 participants) demonstrated that each increase in the BMI of 5 kg/m^2^ was, on average, associated with a higher overall mortality of ≈30% [3].

Recently, several studies have shown obesity to be one of the factors contributing to the development of heart failure in general population [4–6]. According to the data of patients hospitalised with acute heart failure (AHF), development of congestive heart failure occurs earlier in patients who are overweight (BMI 25–30 kg/m^2^) and obese (BMI >30 kg/m^2^) in comparison with patients with normal body weight [7]. Factors that may contribute to the development of heart failure in obese patients include an increased incidence of hypertension (especially diastolic), higher insulin resistance (and the related glucose intolerance and type-2 diabetes mellitus (T2DM)), increased systemic inflammation, and prothrombotic state [8].

Conversely, several studies based on large groups of patients with cardiovascular disease found—contrary to expectations—that overweight and obese patients had a better prognosis compared with patients with normal weight. This phenomenon is called the “obesity paradox”. These studies included patients with AHF [7], with chronic heart failure [9] and after acute myocardial infarction (AMI) [10,11]. The key question is whether the better prognosis is caused by the higher BMI or by other factors that are non-causally associated with the BMI. It is known that overweight/obese patients are younger, receive more aggressive pharmacological intervention, and differ in other characteristics [7,11]. To ascertain if the better prognosis could be related to the higher BMI or to other confounding factors, elimination of the influence of these confounding factors is necessary. Most studies focussing on the obesity paradox have used multivariable logistic regression-based risk adjustment. This approach to elimination of the influence of confounding factors may not be the most appropriate choice in all situations. Another widely used method of adjustment that does not present a problem with different non-overlapping groups is a propensity score [12]. Interestingly, none of the available studies confirmed the hypothesis that overweight/obese patients would have a worse prognosis than patients with normal weight.

Current recommendations set by the European Society of Cardiology (ESC) for the treatment of acute heart failure (AHF) and chronic heart failure (CHF) deal with the risk and therapeutic possibilities of cachexia but, for obesity itself, the ESC recommendations refer to the European guidelines on prevention of cardiovascular disease only. They mention a possible obesity paradox in patients with chronic heart diseases (CHDs), but recommend weight reduction (1A level of recommendation) [13]. Due to a lack of evidence-based medicine about the benefit of this recommendation, we consider the obesity paradox in AHF patients to be a very important and unsolved topic. Therefore, we used a propensity score to assess short-term/long-term mortality rates in patients hospitalised for AHF in the Czech Republic from 2006 to 2012 with respect to the BMI. Subsequently, we focused on patients with acute decompensated heart failure (ADHF) and *de novo* AHF.

## Methods

The study protocol complied with the Declaration of Helsinki, and was approved by the Multicentre Ethics Committee of University Hospital Brno (Brno, Czech Republic). Written informed consent was obtained from all subjects to participate in the study.

### Study population

The Acute Heart Failure Database (AHEAD) Network registry comprises consecutive patients from ten centres with 24-h Catheter Laboratory services and centralised care for patients with acute coronary syndromes (described previously as “AHEAD Main” [14,15]) and from five regional centres without a Catheter Laboratory. The inclusion criteria for the database adhere to the European guidelines for AHF set in 2005: signs and symptoms of AHF; confirmed left-ventricular dysfunction (systolic or diastolic); and/or positive response to therapy [16]. The final diagnosis of acute heart failure was the responsibility of the attending cardiologists, and all consecutive patients who were diagnosed with AHF were enrolled in the registry. Exclusion criteria were: known or newly diagnosed advanced stage of malignancy, disagreement with the participation in the registry.

**Fig 1:**
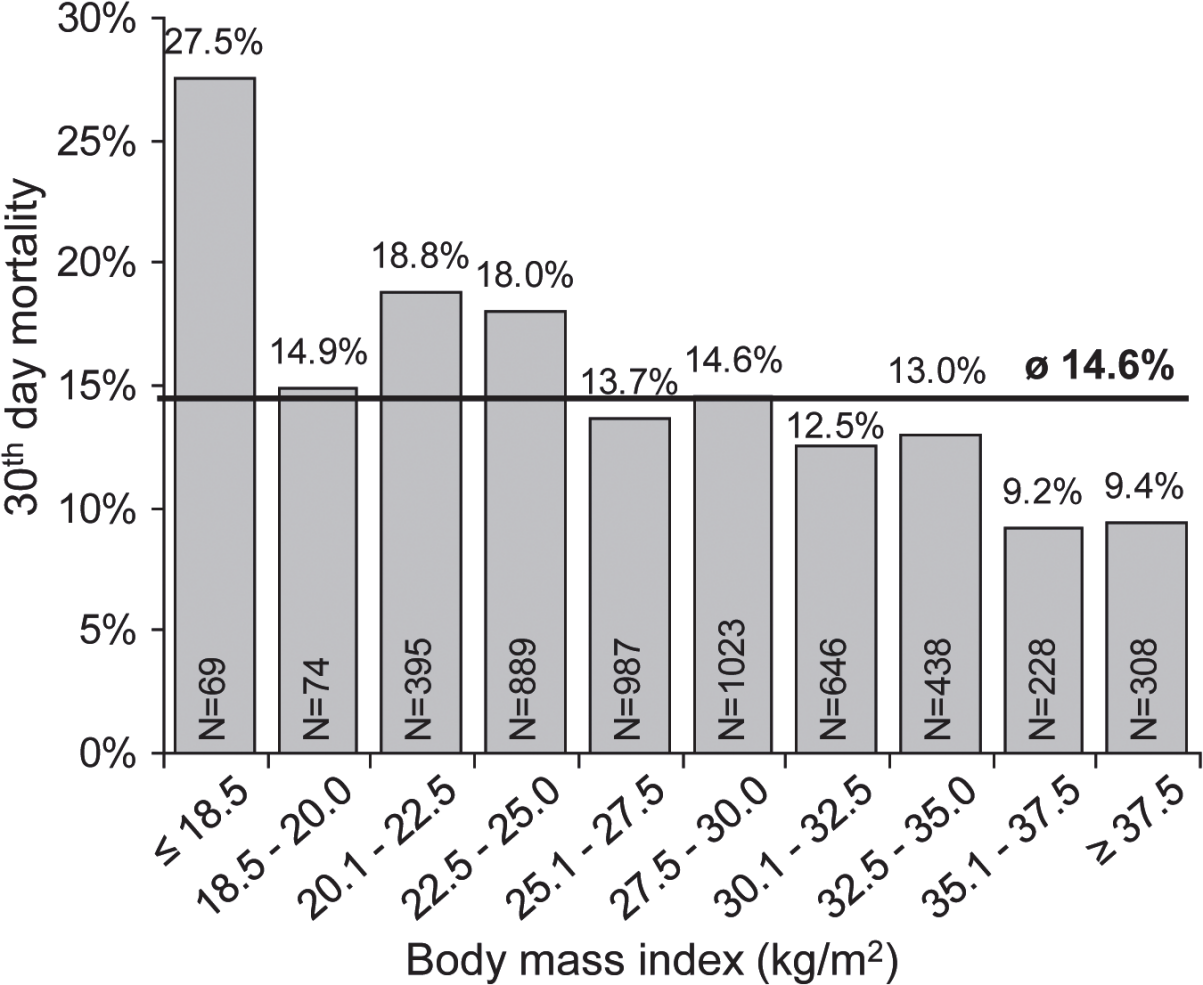
Definition of datasets.

The AHEAD Network registry includes 8818 hospitalisations due to AHF. Data were collected prospectively and evaluated continuously from September 2006 to October 2012 *via* a secured online database accessible from the project website (http://ahead.registry.cz). Stratification of AHF was based on ESC guidelines published in 2005 [16]. The database contains information about concomitant diseases; medication at the time of hospital admission, during hospitalisation, and at hospital discharge; haemodynamic parameters; electrocardiography; biochemistry; echocardiography; invasive procedures. Weight and height were assessed at the time of hospital admission. Only first-hospitalisations (FHs) during the study period were evaluated in the analysis. From the overall number of 6242 FHs, only 5057 records of FHs with information about the BMI were used; 69 patients with a BMI <18.5 kg/m^2^ and probable cachexia were removed from the analysis, as in other studies. Out of 5057 AHF patients, 2228 were analysed using propensity matching. These 2228 patients were subsequently divided into 2 groups: patients with a previous diagnosis of HF (ADHF, N = 978), and those admitted for the first time HF, “*de novo* HF” (N = 1250) (Fig. 1). Assessed patients were divided into two groups according to the BMI: patients with normal weight (BMI = 18.5–25 kg/m^2^) and overweight/obese patients (BMI >25 kg/m^2^). Long-term mortality was followed using the centralised database of death records of the Ministry of Health of the Czech Republic.

### Study outcome

The primary outcome of the analysis was all-cause mortality during follow-up. We focussed on both short-term mortality (30 days after the diagnosis) and long-term mortality, which was monitored via the follow up of surviving patients for at least 2 years (the median follow-up was 32.1 months, ranging from 0 to 77.5 months).

### Statistical analyses

Standard descriptive statistics were applied in the analysis: absolute and relative frequencies for categorical variables and mean supplemented by standard deviation for continuous variables.

Differences in patient characteristics according to the BMI (normal weight, 18–25 kg/m^2^; overweight/obesity >25 kg/m^2^) were tested using the maximum likelihood chi-square test for categorical variables and the independent *t*-test for continuous variables. The propensity score based on logistic regression was used to obtain the balanced dataset. The logistic regression model was constructed based on the Wald test with backward selection. Of the 22 input variables considered prognostically significant according to previous results [14,15], ten non-redundant variables were selected by backward selection (age, hypertension, T2DM, atrial fibrillation, diastolic blood pressure, width of QRS interval, heart rate, haemoglobin, coronary artery disease, acute coronary syndrome upon hospital admission, creatinine).

Thirty-day mortality was expressed as a proportion in patient groups. The influence of normal weight on 30-day mortality was evaluated by an odds ratio (OR) generated by logistic regression; the corresponding statistical significance was evaluated using the Wald test. The Kaplan–Meier estimator was used for the assessment of long-term mortality using censored data. Differences in mortality between patient groups were tested by the log-rank test. The Cox proportional hazards model and hazard ratio (HR) were adopted for analyses of the effect of the BMI on long-term mortality.

The level of significance was set at α = 0.05 for all analyses. IBM SPSS v21 (SPSS, Chicago, IL, USA) and R v2.12.2 (R Foundation for Statistical Computing, Vienna, Austria) with non-random stratification and matching by the propensity score were used for data analyses.

## Results

**Fig 2:**
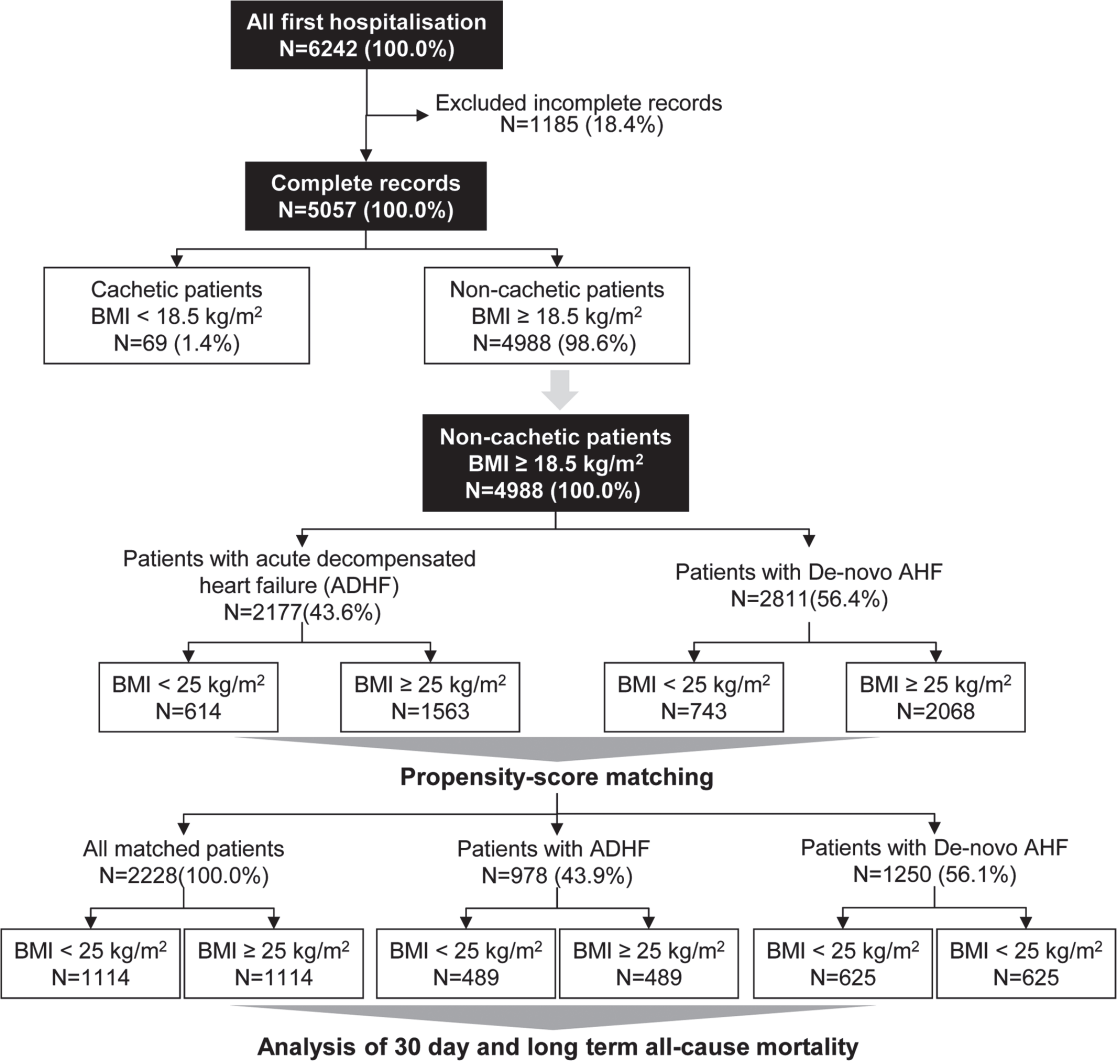
Thirty-day mortality according to BMI category.

A total of 5057 patients were assessed. The distribution of patients according to their BMI and 30-day mortality is shown in Fig. 2. Mean 30-day mortality in the entire cohort was 14.6%; the highest mortality (27.5%) was observed in the subgroup of patients with a BMI <18.5 kg/m^2^, these patients were excluded from subsequent analyses (Fig. 2).

In 4988 patients with a BMI ≥18.5 kg/m^2^ that were evaluated, 43.6% of these patients were admitted for ADHF and 56.4% of patients for *de novo* heart failure (HF). The patients with normal weight (BMI, 18.5–25 kg/m^2^) were significantly different in comparison with overweight/obese patients (BMI >25 kg/m^2^). Patients with normal weight were older (>70 years, 68.4% *vs* 55.7%, p<0.001); were more often women (45.0% *vs* 40.3%, p = 0.003). Upon hospital admission, patients with normal weight had lower systolic blood pressure (<100 mmHg, 15.5% *vs* 12.2%, p = 0.003), lower haemoglobin level (<120 g/l, 67.8% *vs* 74.9%, p<0.001), and were less likely to have T2DM (31.8% *vs* 50.0%, p<0.001) and hypertension (67.6% *vs* 77.5%, p<0.001) (Table 1). Baseline values of brain natriuretic peptide (BNP) were comparable in both groups. However, baseline values of N-terminal of the prohormone brain natriuretic peptide (NT-proBNP) as well as maximal values of BNP and NT-proBNP during hospitalisation were higher in patients with normal weight. Due to the high proportion of missing data, natriuretic peptides were not included in the propensity score. Upon hospital admission, more overweight/obese patients had previous treatment by ACEI or ARB (70.1% *vs* 64.5%; p <0.001) and beta-blockers (54.1% vs 47.6%; p<0.001). More overweight/obese patients had coronary angiography examination in comparison with patients with normal weight.

**Table 1:** Baseline characteristics of patients before and after matching.

Before matching					After matching		
	Valid N	Total	BMI 18.5–25	BMI >25	Valid N	BMI 18.5–25	BMI >25
		N (%)/ mean ± SD	N (%)/ mean ± SD	N (%)/ mean ± SD		N (%)/ mean ± SD	N (%)/ mean ± SD
N		4988	1357	3631		1114	1114
Sex (females)	4988	41.6%	45.0%	40.3%*	2228	44.9%	44.3%
Age (at admission)^a^	4988	72 ± 12	74 ± 14	71 ± 11**	2228	73 ± 14	73 ± 11
BMI (kg/m^2^)	4985	28.9 ± 9.1	23.0 ± 1.8	31.0 ± 9.8**	2225	23.1 ± 1.8	30.5 ± 8.8**
Systolic BP (mmHg)	4966	138 ± 34	135 ± 34	140 ± 34**	2228	137 ± 34	136 ± 33**
Diastolic BP (mmHg) ^a^	4966	81 ± 18	78 ± 18	81 ± 18**	2228	80 ± 18	79 ± 18**
Heart rate (min^-1^) ^a^	4965	93 ± 27	94 ± 27	92 ± 27	2228	93 ± 26	93 ± 27*
Width of QRS (ms) ^a^	4208	106 ± 28	108 ± 29	105 ± 28*	1885	107 ± 29	107 ± 29
LVEF (%)	2012	38.1 ± 14.2	37.1 ± 14.5	38.5 ± 14.1	843	37.2 ± 14.6	38.0 ± 14.6*
Chronic NYHA III/IV	4845	44.4%	44.5%	44.4%	2218	44.7%	45.7%
*Medical history*							
Atrial fibrillation^+^	4952	28.3%	27.8%	28.5%	2228	29.0%	28.0%
Hypertension^+^	4988	74.8%	67.6%	77.5%**	2228	71.4%	70.2%
Diabetes mellitus^+^	4883	45.1%	31.8%	50.0%**	2228	35.6%	35.1%
History of MI	4867	31.7%	29.9%	32.3%	2227	30.7%	29.8%
History of stroke/TIA	4869	17.2%	19.4%	16.3%*	2226	19.8%	18.3%
History of PCI or CABG	4869	18.7%	15.9%	19.8%*	2228	16.8%	16.9%
History of PM/ICD/CRT	4981	13.1%	13.7%	12.9%	2228	13.8%	13.2%
Coronary artery disease ^a^	4988	55.5%	53.7%	56.1%	2228	54.2%	53.8%
COPD	4867	19.1%	18.8%	19.2%	2227	18.2%	21.5%
Coronary angiography	4986	44.2%	38.3%	46.4%**	2228	39.9%	43.4%
*Current AHF*							
Pulmonary oedema	4987	19.3%	19.7%	19.1%	2227	19.7%	18.0%
Cardiogenic shock	4987	12.2%	14.5%	11.3%*	2227	12.7%	10.8%
Acute coronary syndrome	4988	35.0%	33.8%	35.5%	2228	33.8%	35.0%
*Laboratory values at admission*							
Creatinine (μmol/l) ^a^	4988	125 ± 73	126 ± 85	125 ± 68	2228	128 ± 88	122 ± 66
Sodium (mmol/l)	4925	138 ± 4	138 ± 4	138 ± 4	2225	138 ± 4	138 ± 4
Potassium (mmol/l)	4922	4.21 ± 0.66	4.20 ± 0.66	4.22 ± 0.67	2222	4.20 ± 0.64	4.21 ± 0.68
Glucose (mmol/l)	4804	9.69 ± 6.89	9.43 ± 8.03	9.78 ± 6.43	2173	9.51 ± 7.90	9.37 ± 7.07
Cholesterol (mmol/l)	4988	4.42 ± 1.28	4.38 ± 1.18	4.44 ± 1.31	2228	4.40 ± 1.20	4.49 ± 1.29*
Triacylglycerides (mmol/l)	1538	1.45 ± 1.04	1.23 ± 0.89	1.53 ± 1.07**	699	1.22 ± 0.85	1.39 ± 0.98**
Haemoglobin (g/l) ^a^	4855	131 ± 20	128 ± 20	132 ± 20**	2228	130 ± 19	130 ± 20**
BNP (pg/ml)^b^	635	1519 ± 1891	1675 ± 1712	1467 ± 1946	280	1673 ± 1660	1560 ± 2021
BNP max. (pg/ml) ^b^	312	1885 ± 2128	2343 ± 2170	1708 ± 2090*	152	2304 ± 2107	1700 ± 2079*
NTproBNP (pg/ml) ^b^	1021	8357 ± 8 194	10600 ± 9571	7476 ± 7409**	473	10309 ± 9 14	7848 ± 7463**
NTproBNP max (pg/ml)^b^	502	9844 ± 10 021	12704 ± 11140	8813 ± 9391**	237	12871 ± 11119	9154 ± 9426**
*Medication at* discharge^c^							
ACE inhibitor/ARB	4497	80.1%	75.3%	81.8%**	2129	76.3%	78.1%
Betablockers	4688	77.1%	73.5%	78.4%**	2129	74.1%	70.7%
Diuretics	4687	83.6%	84.0%	83.4%	2129	83.6%	86.2%

*/**Statistical significance of differences between groups was tested by the ML chi-square test for categorical variables and by the independent Student’s *t*-test for continuous variables

*/**p<0.05/p<0.001

^a^Parameter used in a logistic regression model of a propensity score

^b^Parameter was not known for all patients, and statistics were computed on a reduced basis

^c^Medication at discharge was computed on patients who were alive after discharge

LVEF—left ventricular ejection fraction, BP—blood pressure, MI—myocardial infarction, TIA—transient ischemic attack, PCI—percutaneous coronary intervention, CABG—coronary artery bypass graft, PM—pacemaker, ICD—implantable cardioverter–defibrillator, CRT—cardiac resynchronization therapy, COPD—chronic obstructive pulmonary disease, ARB—angiotensin-2 receptor blockers.

**Fig 3:**
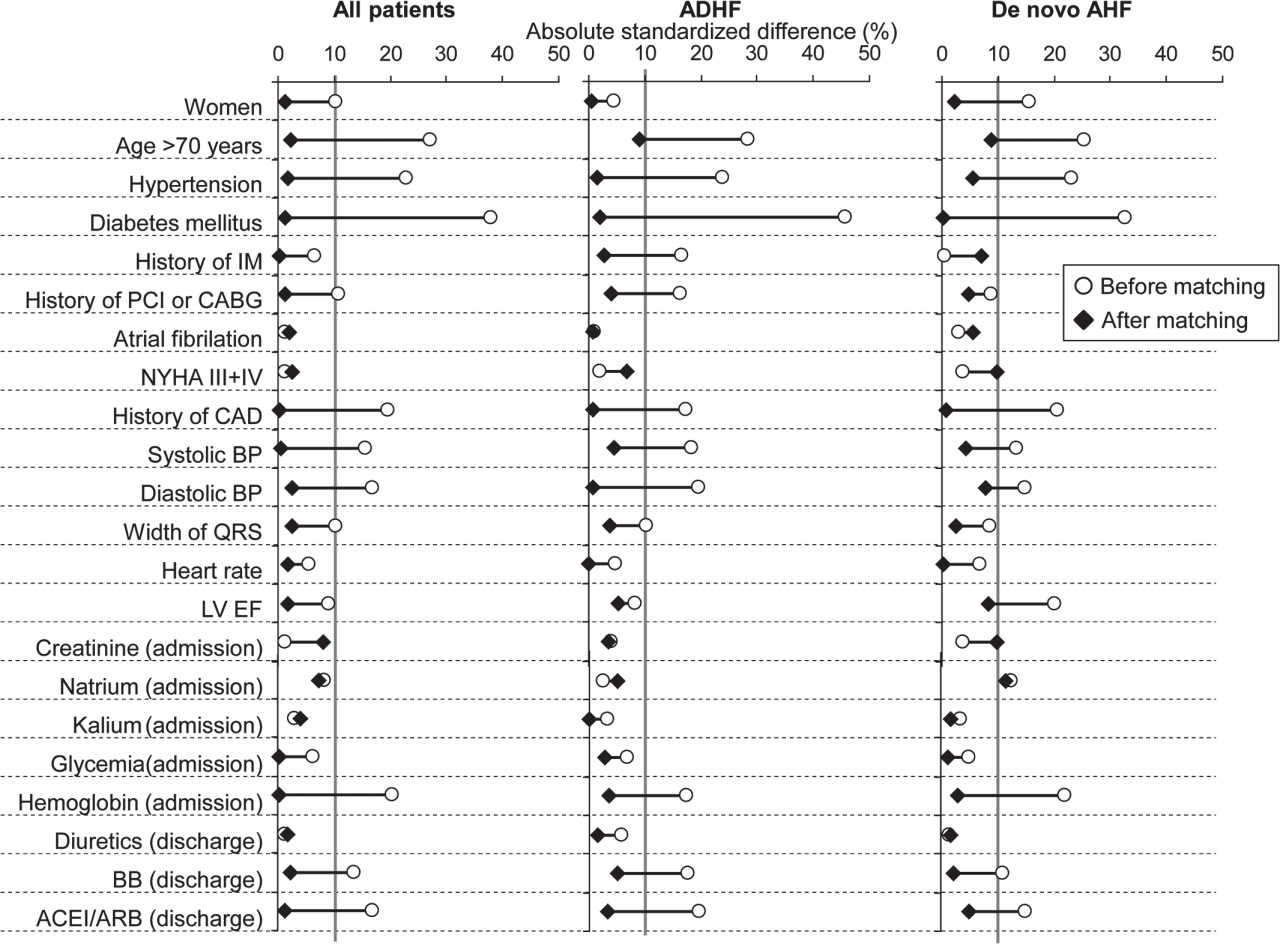
Absolute standardized differences (%) in observed covariates between patients with normal weight and those who were overweight/obese before and after matching of propensity score (age, diastolic blood pressure, heart rate, width of QRS interval, atrial fibrillation, hypertension, type-2 diabetes mellitus, coronary artery disease, creatinine level and haemoglobin level). Covariates with a post-match absolute standardized difference <10% were considered successfully balanced.

Using a propensity score based on ten parameters that probably affect the prognosis, a balanced dataset according to a BMI of 25 kg/m^2^ was created (Fig. 3). The balanced dataset contained 2228 patients (1114 patients for each group). Baseline characteristics are detailed in Table 1. Balanced datasets in subgroups of patients with ADHF and *de novo* HF patients were prepared using the same approach (Table 2). After balancing, the standardised differences in groups according to the BMI were <10% for most of the parameters.

**Table 2:** Baseline characteristics of patients after matching according to type of heart failure.

	Decompensated heart failure		*De novo* heart failure	
	BMI 18.5–25	BMI >25	BMI 18.5–25	BMI >25
	N (%) mean ± SD	N (%) mean ± SD	N (%) mean ± SD	N (%) mean ± SD
N	489	489	625	625
Sex (females)	40.9%	41.1%	48.0%	46.9%
Age at admission^a^	75 ± 13	74 ± 10	72 ± 14	71 ± 11
BMI (kg/m^2^)	23.1 ± 2.1	30.4 ± 9.0**	23.1 ± 1.6	30.7 ± 8.5**
Systolic BP (mmHg)	135 ± 32	136 ± 31	138 ± 35	137 ± 34
Diastolic BP (mmHg) ^a^	79 ± 16	79 ± 17	80 ± 20	79 ± 19
Heart rate (min^-1^) ^a^	92 ± 24	92 ± 25	95 ± 27	95 ± 29
Width of QRS (ms) ^a^	115 ± 31	114 ± 32	102 ± 27	101 ± 26
LVEF (%)	35.6 ± 15.6	33.3 ± 14.8	38.0 ± 14.1	40.3 ± 14.0
Chronic NYHA III/IV	67.8%	64.6%	26.6%	31.0%
*Medical history*				
Atrial fibrillation^a^	35.2%	32.5%	24.2%	24.5%
Hypertension^a^	76.5%	77.1%	67.4%	64.8%
Diabetes mellitus^a^	40.5%	39.5%	31.8%	31.7%
History of MI	44.6%	46.0%	19.8%	17.1%
History of stroke/TIA	22.1%	21.6%	18.1%	15.7%
History of PCI or CABG	27.6%	29.4%	8.3%	7.0%
History of PM/ICD/CRT	22.9%	24.9%	6.7%	4.0%*
Coronary artery disease ^a^	51.9%	51.7%	56.0%	55.4%
COPD	24.9%	27.8%	13.0%	16.5%
Coronary angiography	20.0%	24.9%	55.4%	57.9%
*Syndrome of AHF*				
Pulmonary oedema	19.2%	18.4%	20.2%	17.6%
Cardiogenic shock	7.8%	6.3%	16.6%	14.3%
Acute coronary syndrome	15.3%	16.0%	48.3%	49.9%
*Laboratory values at admission*				
Creatinine (umol/l) ^a^	134 ± 72	132 ± 70	123 ± 99	114 ± 61
Sodium (mmol/l)	138 ± 4	138 ± 5	138 ± 5	138 ± 4
Potassium (mmol/l)	4.28 ± 0.64	4.28 ± 0.69	4.15 ± 0.64	4.16 ± 0.66
Glucose (mmol/l)	8.84 ± 7.25	8.66 ± 5.76	10.0 ± 8.3	9.92 ± 7.89
Cholesterol (mmol/l)	3.97 ± 1.11	4.12 ± 1.25	4.68 ± 1.17	4.75 ± 1.25
Triacylglycerides (mmol/l)	1.06 ± 0.51	1.24 ± 0.77*	1.29 ± 0.95	1.47 ± 1.06
Haemoglobin (g/l) ^a^	127 ± 19	128 ± 20	131 ± 19	132 ± 20
*Medication at discharge* ^*b*^				
ACE inhibitor/ARB	77.3%	78.6%	75.6%	77.7%
Beta blockers	73.8%	76.0%	74.3%	73.3%
Diuretics	92.6%	93.0%	76.1%	75.4%

*/**Statistical significance of differences between groups tested by the ML chi-square test for categorical variables and by the independent Student’s *t*-test for continuous variables

*/**p<0.05/p<0.001

^a^Parameters used in a logistic regression model of a propensity score

^b^Medication at discharge was computed on patients who were alive after discharge

LVEF—left ventricular ejection fraction, BP—blood pressure, MI—myocardial infarction, TIA—transient ischemic attack, PCI—percutaneous coronary intervention, CABG—coronary artery bypass graft, PM—pacemaker, ICD—implantable cardioverter–defibrillator, CRT—cardiac resynchronization therapy, COPD—chronic obstructive pulmonary disease, ARB—angiotensin-2 receptor blockers.

**Fig 4:**
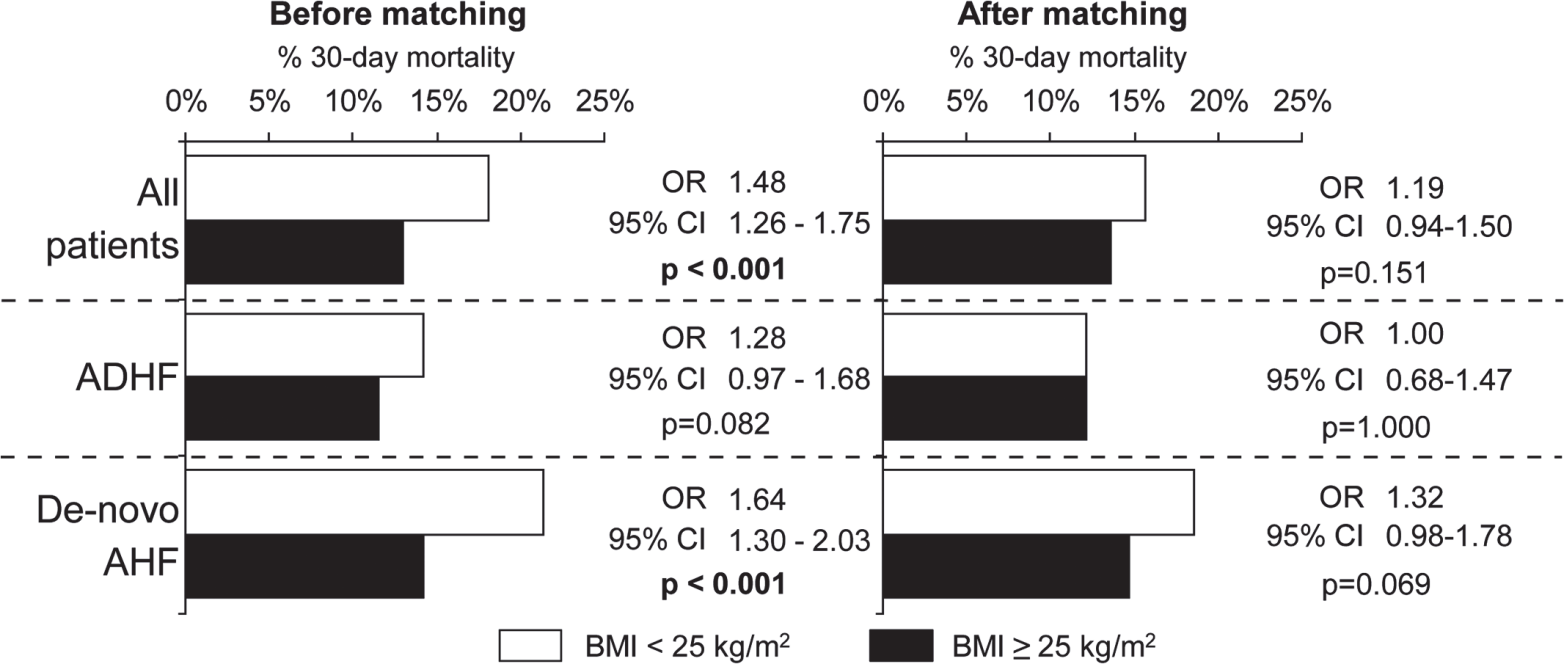
Thirty-day mortality according to the BMI category and type of heart failure.

In the unbalanced dataset, 30-day mortality was significantly higher in patients with normal weight in comparison with overweight/obese patients (18.1% *vs* 13.0%; OR, 1.48; 95% CI, 1.26–1.75; p<0.001) (Fig. 4). Similar trends were observed in the subgroups of patients with ADHF and *de novo* AHF but the difference was significant only for *de novo* AHF. In balanced groups, significant differences in 30-day mortality were not observed (Fig. 4).

**Fig 5:**
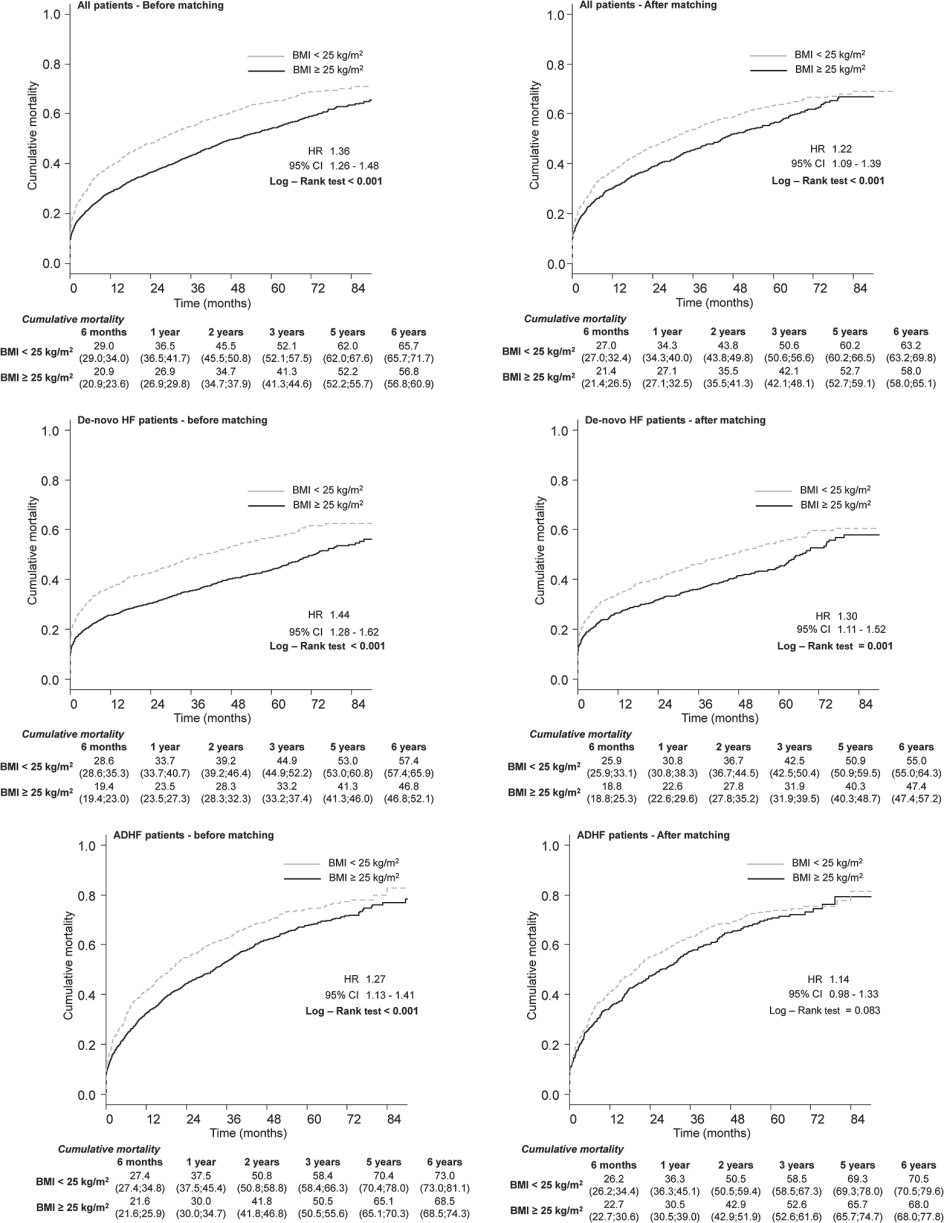
Long-term mortality for all patients according to a BMI of 25 kg/m^2^ in a non-balanced and balanced dataset and for ADHF and *de novo* AHF patients.

Kaplan–Meier curves for the unbalanced dataset (Fig. 5) revealed the long-term mortality of patients with normal weight to be higher than for those who were overweight/obese (HR, 1.36; 95% CI, 1.26–1.48; p<0.001). An identical pattern was observed in the balanced dataset (HR, 1.22; 95% CI, 1.09–1.39; p<0.001)) (Fig. 5). Similarly, long-term mortality in the subgroup of patients with *de novo* AHF was higher for patients with normal weight in the unbalanced dataset (HR, 1.44; 95% CI, 1.28–1.62; p<0.001)) (Fig. 5) as well as in the balanced dataset (1.30; 1.11–1.52; 0.001) (Fig. 5). In the unbalanced dataset of patients with ADHF, long-term mortality of patients with normal weight was higher in comparison with overweight/obese patients (HR, 1.27; 95% CI, 1.13–1.41; p<0.001) (Fig. 5), whereas the difference in long-term mortality was not significant in the balanced dataset (1.14; 0.98–1.33; 0.083) (Fig. 5).

## Discussion

We demonstrated the obesity paradox in a large cohort of patients hospitalised for AHF with a median follow-up of 32.1 (range, 0–77.5) months. Until now, studies have followed only hospital, 30-day, or one-year mortality in AHF patients [17]. The obesity paradox was clearly present for long-term mortality in balanced groups of all patients with AHF, as well as in balanced groups of patients with *de novo* AHF. Only a statistically non-significant trend was found in balanced groups of patients with ADHF. At 30 days, the mortality was similar when comparing the balanced groups of all patients with AHF and patients with ADHF. In patients with *de novo* AHF, a trend of lower mortality in balanced groups of overweight/obese patients was observed. We suggest three reasons for the obesity paradox, as discussed below.

The first potential reason is cardiac cachexia. Cachexia has been demonstrated to be an independent negative prognostic factor in patients with advanced HF [18,19]. Patients with a BMI <18.5 kg/m^2^ were excluded from our analyses because of the supposed presence of cardiac cachexia. A similar methodological approach for the evaluation of patients with AMI complicated by HF was applied in a recent study [20]. Conversely, according to the latest definition, cachexia might be assumed even in patients with a BMI <20 kg/m^2^ [21]. This cachexia could be one of the factors that contribute to obesity paradox.

Cachexia is defined as weight loss of 5% in the previous 12 months accompanied by decreased muscle strength, fatigue loss of appetite, low fat-free mass index or abnormal biochemistry (including increased inflammatory markers, anaemia (<120 g/l) or low serum albumin (<32 g/l)). We can assume that cardiac cachexia could have occurred in patients who were initially overweight or obese and who, due to cachexia, moved into the category of patients with normal weight. This assumption is supported by the findings of Melenovsky et al., who diagnosed cachexia by the criteria stated above in 19% of cases with chronic HF, whereas only 2% of them had a BMI <20 kg/m^2^ [18] Of the parameters appropriate for the auxiliary laboratory diagnosis of cachexia, we observed only haemoglobin in our dataset. In the group of patients with normal weight, 32.2% had a haemoglobin level <120 g/l in comparison with 25.1% in the group of overweight/obese patients (p<0.001). This result supports the possible participation of cachexia in the obesity paradox in patients hospitalised for AHF.

The second potential reason is the metabolic activity of adipose tissue. Adipose tissue (particularly visceral fat) is metabolically active and produces/influences the production of cytokines associated with inflammation and oxidative stress [22]. Some adipocytokines (e.g., resistin, adiponectin, visfatin) may have cardioprotective effects. These adipocytokines have been demonstrated to be involved in reduction of infarct size and apoptosis in a cardiac model of ischaemia–reperfusion injury [23–25].

The final reason is metabolic and energy reserves. ADHF is often accompanied by loss of appetite. HF patients are often also re-hospitalised for reasons other than ADHF, and it has been demonstrated that hospitalisation in the intensive care unit is habitually associated with malnutrition [26]. Obese patients can better tolerate such temporary malnutrition and use their energy reserves. It can be assumed that obese patients can better utilise energy and nutrients from ingested food, which could be a protective factor in the case of impaired intake of food and increased metabolic demands.

Our results are consistent with a comprehensive analysis of the Acute Decompensated Heart Failure National Registry (ADHERE) database, which evaluated 108,927 patients hospitalised due to ADHF, and which clearly demonstrated higher mortality to be associated with the lowest quartile of the BMI. In that study, patients with the lowest quartile of the BMI had the lowest left-ventricular ejection fraction (LVEF) and higher level of NT-proBNP compared with patients with a higher BMI even after adjustment for age and other parameters [7]. We included LVEF as one of the prognostic parameters used to create the balanced group using a propensity score and, therefore, LVEF values were balanced between groups (LVEF 38.0±14.6% *vs* 37.2±14.6%). Values of natriuretic peptides (with the exception of BNP upon hospital admission) were significantly higher in the group with a BMI 18.5–25 kg/m^2^ compared with overweight/obese patients (Table 1). Due to the limited number of patients for whom values of BNP/NT-proBNP were available, natriuretic peptides were not included in our model for the balancing process. However, one study clearly demonstrated levels of natriuretic peptides to be inversely correlated with the BMI [27], and that BNP/NT-proBNP levels were lower in obese patients [28]. Cachectic patients had twofold higher levels of natriuretic peptides in comparison with haemodynamically comparable patients who did not have cachexia [18]. Therefore, we believe that, despite the lower levels of natriuretic peptides in overweight/obese patients, both groups were comparable with regard to severity of HF, and that the level of natriuretic peptides is not a suitable parameter for comparison of HF severity among groups with different BMI values.

The obesity paradox in patients with AHF was confirmed in a recent study that used logistic regression analyses to adjust for differences in risk factors between BMI groups. A higher BMI continued to be associated with decreased 30-day and 1-year mortality (11% decrease at 30 days; 9% decrease at 1 year per 5 kg/m^2^ [17].

Our work demonstrated that patients with a higher BMI in the unbalanced primary dataset were younger and had differences similar to those reported previously [9,17]. An important aspect that could affect the results is selection of appropriate statistical analyses: two approaches are possible. Adjustment by propensity-score matching and by conventional regression methods differ fundamentally in their approach to investigation of treatment/exposure effects. Matching can eliminate a greater proportion of bias and then estimate the effect of the analysed group more precisely as compared with other approaches; the result of matching is a balanced dataset that allows a simple and direct comparison of baseline covariates between groups of patients. Conversely, a balanced dataset obtained by matching does not contain all patients, so some information about the structure of the original dataset is lost. Adjustment by logistic regression analyses eliminates the influence of bias by using a logistic regression equation, and does not lose such information as propensity-score matching does. However, several assumptions must be met for valid results, and adjustment by logistic regression modifies the values of cases in non-overlapping areas of the dataset. A propensity score excludes these areas from the analysis, and thus elicits less bias than regression adjustment [29].

The present study had several limitations. Firstly, some patients had an overestimated BMI value due to fluid retention upon hospital admission (when the weight was established). Swelling of the extremities was observed in 35.2% of patients with a BMI 18.5–25 kg/m^2^ and in 42.0% of patients with a BMI >25 kg/m^2^. Five-year all-cause mortality in our patients with swelling was significantly higher in comparison with those without swelling (44.9% vs 33.4%; p<0.001). This supports the concept of the obesity paradox because patients with a higher BMI due to swelling (and the higher prevalence of mortality of such patients) could move into the group with normal weight after weight correction. Secondly, only all-cause mortality was monitored instead of cardiovascular mortality, which could have revealed more information about the relationship between obesity and HF. Thirdly, patients with advanced cancer were not included in the database, but it can be assumed that a very small proportion of patients could be in cancer remission or that cancer was not diagnosed by the time of hospitalisation. Fourthly, the methodology applied for balancing datasets can lead to removal of non-overlapping extreme cases (i.e., the balanced dataset is not fully representative of primary unbalanced data). Lastly, we evaluated only the BMI and no other parameters that might be more closely associated with visceral fat and its metabolic activity. For example, besides the BMI, the Visceral Adiposity Index (VAI) also includes waist circumference as well as levels of high-density lipoprotein-cholesterol (HDL-C) and triglycerides. Waist circumference provides information about the amount of visceral fat, but levels of HDL-C and triglycerides provide information about its activity. Recently, it was demonstrated that VAI (unlike BMI or waist circumference) is an independent prognostic predictor of increased cerebrovascular and cardiovascular morbidity [30].

## Conclusion

We clearly demonstrated that, although overweight/obese patients hospitalised due to AHF were younger in comparison with patients with a normal BMI, their long-term prognosis was significantly better. This effect could be observed even after creation of a balanced dataset using a propensity score. In particular, these results are valid for patients with *de novo* AHF. This work could form the basis of an investigation of the pathophysiological mechanisms of the obesity paradox and prospective intervention studies to confirm the improved prognosis in overweight/obese patients with HF.

## References

[1] Berrington de Gonzalez A, Hartge P, Cerhan JR, Flint AJ, Hannan L et al (2010) Body-mass index and mortality among 1.46 million white adults. N Engl J Med 363: 2211–19. 10.1056/NEJMoa1000367 21121834 PMC3066051

[2] Zheng W, McLerran DF, Rolland B, Zhang X, Inoue M et al (2011) Association between Body-Mass Index and Risk of Death in More Than 1 Million Asians. N Engl J Med 364: 719–29. 10.1056/NEJMoa1010679 21345101 PMC4008249

[3] Prospective Studies Collaboration (2009) Body-mass index and cause-specific mortality in 900 000 adults: collaborative analyses of 57 prospective studies. Lancet 373: 1083–96. 10.1016/S0140-6736(09)60318-4 19299006 PMC2662372

[4] He J, Ogden LG, Bazzano LA, Vupputuri S, Loria C et al (2001) Risk factors for congestive heart failure in US men and women: NHANES I epidemiologic follow-up study. Arch Intern Med 161: 996–1002. 11295963 10.1001/archinte.161.7.996

[5] Kenchaiah S, Evans JC, Levy D, Wilson PW, Benjamin EJ et al (2002) Obesity and the risk of heart failure. N Engl J Med 347: 305–13. 12151467 10.1056/NEJMoa020245

[6] Wilhelmsen L, Rosengren A, Eriksson H, Lappas G (2001) Heart failure in the general population of men—morbidity, risk factors and prognosis. J Intern Med 249: 253–61. 11285045 10.1046/j.1365-2796.2001.00801.x

[7] Fonarow GC, Srikanthan P, Costanzo MR, Cintron GB, Lopatin M (2007) An obesity paradox in acute heart failure: analysis of body mass index and inhospital mortality for 108,927 patients in the Acute Decompensated Heart Failure National Registry. Am Heart J 153: 74–81. 17174642 10.1016/j.ahj.2006.09.007

[8] Poirier P, Giles TD, Bray GA, Hong Y, Stern JS et al (2006) Obesity and cardiovascular disease: pathophysiology, evaluation, and effect of weight loss: an update of the 1997 American Heart Association Scientific Statement on Obesity and Heart Disease from the Obesity Committee of the Council on Nutrition, Physical Activity, and Metabolism. Circulation 113: 898–918. 16380542 10.1161/CIRCULATIONAHA.106.171016

[9] Curtis JP, Selter JG, Wang Y, Rathore SS, Jovin IS et al (2005) The obesity paradox: body mass index and outcomes in patients with heart failure. Arch Intern Med 165: 55–61. 15642875 10.1001/archinte.165.1.55

[10] Diercks DB, Roe MT, Mulgund J, Pollack CV Jr, Kirk JD et al (2006) The obesity paradox in non-ST-segment elevation acute coronary syndromes: results from the Can Rapid risk stratification of Unstable angina patients Suppress ADverse outcomes with Early implementation of the American College of Cardiology/American Heart Association Guidelines Quality Improvement Initiative. Am Heart J 152: 140–8. 16824844 10.1016/j.ahj.2005.09.024

[11] Angerås O, Albertsson P, Karason K, Ramunddal T, Matejka G et al (2013) Evidence for obesity paradox in patients with acute coronary syndromes: a report from the Swedish Coronary Angiography and Angioplasty Registry. Eur Heart J 34: 345–53. 10.1093/eurheartj/ehs217 22947610

[12] Frankenstein L, Zugck C, Nelles M, Schellberg D, Katus HA et al (2009) The obesity paradox in stable chronic heart failure does not persist after matching for indicators of disease severity and confounders. Eur J Heart Fail 11: 1189–94. 10.1093/eurjhf/hfp150 19887494

[13] Perk J, Backer GD, Gohlke H, Graham I, Reiner Z et al (2012) European Guidelines on cardiovascular disease prevention in clinical practice (version 2012) The Fifth Joint Task Force of the European Society of Cardiology and Other Societies on Cardiovascular Disease Prevention in Clinical Practice (constituted by representatives of nine societies and by invited experts) Developed with the special contribution of the European Association for Cardiovascular Prevention & Rehabilitation (EACPR). Eur Heart J 33: 1653–701. 10.1093/eurheartj/ehs092 22555213

[14] Spinar J, Parenica J, Vitovec J, Widimsky P, Linhart A et al (2011) Baseline characteristics and hospital mortality in the Acute Heart Failure Database (AHEAD) Main registry. Crit Care 15: R291 10.1186/cc10584 22152228 PMC3388663

[15] Parenica J, Spinar J, Vitovec J, Widimsky P, Linhart A et al (2013) Long-term survival following acute heart failure: the Acute Heart Failure Database Main registry (AHEAD Main). Eur J Intern Med 24: 151–60. 10.1016/j.ejim.2012.11.005 23219321

[16] Nieminen MS, Böhm M, Cowie MR, Dexler H, Filippators GS et al (2005) Executive summary of the guidelines on the diagnosis and treatment of acute heart failure The Task Force on Acute Heart Failure of the European Society of Cardiology. Eur Heart J 26: 384–416. 15681577 10.1093/eurheartj/ehi044

[17] Shah R, Gayat E, Januzzi JL, Sato N, Cohen-Solal A et al (2014) Body mass index and mortality in acutely decompensated heart failure across the world: a global obesity paradox. J Am Coll Cardiol 63: 778–85. 10.1016/j.jacc.2013.09.072 24315906

[18] Melenovsky V, Kotrc M, Borlaug BA, Marke T, Kovar J et al (2013) Relationships between right ventricular function, body composition, and prognosis in advanced heart failure. J Am Coll Cardiol 62: 1660–70. 10.1016/j.jacc.2013.06.046 23916933

[19] Anker SD, Ponikowski P, Varney S, Chua TP, Clark AL et al (1997) Wasting as independent risk factor for mortality in chronic heart failure. Lancet 349: 1050–3. 9107242 10.1016/S0140-6736(96)07015-8

[20] Wu AH, Pitt B, Anker SD, Vincent J, Mujib M et al (2010) Association of obesity and survival in systolic heart failure after acute myocardial infarction: potential confounding by age. Eur J Heart Fail 12: 566–73. 10.1093/eurjhf/hfq043 20354030 PMC2875183

[21] Evans WJ, Morley JE, Argilés J, Bales C, Baracos V et al (2008) Cachexia: a new definition. Clin Nutr Edinb Scotl 27: 793–9. 10.1016/j.clnu.2008.06.013 18718696

[22] Musaad S, Haynes EN (2007) Biomarkers of obesity and subsequent cardiovascular events. Epidemiol Rev 29: 98–114. 17494057 10.1093/epirev/mxm005 PMC4682894

[23] Gao J, Chang Chua C, Chen Z, Wang H, Xu X et al (2007) Resistin, an adipocytokine, offers protection against acute myocardial infarction. J Mol Cell Cardiol 43: 601–9. 17904155 10.1016/j.yjmcc.2007.08.009 PMC2692307

[24] Tao L, Gao E, Jiao X, Yuan Y, Li S et al (2007) Adiponectin cardioprotection after myocardial ischemia/reperfusion involves the reduction of oxidative/nitrative stress. Circulation 115: 1408–16. 17339545 10.1161/CIRCULATIONAHA.106.666941

[25] Lim SY, Davidson SM, Paramanathan AJ, Smith CCT, Yellon DM et al (2008) The novel adipocytokine visfatin exerts direct cardioprotective effects. J Cell Mol Med 12: 1395–403. 10.1111/j.1582-4934.2008.00332.x 18400051 PMC2905617

[26] Corkins MR, Guenter P, Dimaria-Ghalili RA, Jensen GL, Malone A et al (2013) Malnutrition Diagnoses in Hospitalized Patients: United States, 2010. J Parenter Enteral Nutr 38: 186–95 10.1177/0148607113512154 24247093

[27] Iwanaga Y, Kihara Y, Niizuma S, Noguchi T, Nonogi H et al (2007) BNP in overweight and obese patients with heart failure: an analysis based on the BNP-LV diastolic wall stress relationship. J Card Fail 13: 663–7. 17923359 10.1016/j.cardfail.2007.05.002

[28] Horwich TB, Hamilton MA, Fonarow GC (2006) B-type natriuretic peptide levels in obese patients with advanced heart failure. J Am Coll Cardiol 47: 85–90. 16386669 10.1016/j.jacc.2005.08.050

[29] Biondi-Zoccai G, Romagnoli E, Agostoni P, Capodanno D, Castagno D et al (2011) Are propensity scores really superior to standard multivariable analysis? Contemp Clin Trials 32: 731–40. 10.1016/j.cct.2011.05.006 21616172

[30] Amato MC, Giordano C, Galia M, Criscimanna A, Vitabile S et al (2010) Visceral Adiposity Index: a reliable indicator of visceral fat function associated with cardiometabolic risk. Diabetes Care 33: 920–2. 10.2337/dc09-1825 20067971 PMC2845052

